# Post-COVID-19 condition risk in patients with intellectual and developmental disabilities: a retrospective cohort study involving 36,308 patients

**DOI:** 10.1186/s12916-023-03216-8

**Published:** 2023-12-20

**Authors:** Ting-Hui Liu, Po-Yu Huang, Jheng-Yan Wu, Min-Hsiang Chuang, Wan-Hsuan Hsu, Ya-Wen Tsai, Pei-Hsin Kao, Chih-Cheng Lai

**Affiliations:** 1 Department of Psychiatry, Chi Mei Medical Center, No 901, Chunghwa Road, Yongkang District, Tainan City 710, Taiwan; 2 Department of Internal Medicine, Chi Mei Medical Center, Tainan, Taiwan; 3 Department of Nutrition, Chi Mei Medical Center, Tainan, Taiwan; 4 Center for Integrative Medicine, Chi Mei Medical Center, Tainan, Taiwan; 5 Department of Geriatric Psychiatry, Chi-Mei Medical Center, Tainan, Taiwan; 6 Division of Hospital Medicine, Department of Internal Medicine, Chi Mei Medical Center, Tainan, Taiwan; 7 School of Medicine, College of Medicine, National Sun Yat-Sen University, Kaohsiung, Taiwan

**Keywords:** COVID-19, Intellectual and developmental disability, Post-COVID-19 condition, SARS-CoV-2

## Abstract

**Background:**

To date, no studies have investigated the prevalence of post-COVID-19 conditions in patients with Intellectual and Developmental Disabilities (IDD). Addressing this research gap is crucial, as understanding post-COVID-19 conditions in IDD patients can improve care planning, and it is essential not to overlook this vulnerable population in COVID-19 studies. This study was aimed at investigating the prevalence of post-COVID-19 conditions in patients with IDD and compare their risk with that of the general population.

**Methods:**

Using the TriNetX network, we identified patients with and without an IDD who had COVID-19. Subsequently, we compared the risk of developing any post-COVID-19 condition between these two groups, during the 90–180-day follow-up after SARS-CoV-2 infection.

**Results:**

During the follow-up, patients with an IDD exhibited a significantly higher prevalence of post-COVID-19 conditions compared to the general population (hazard ratio [HR], 1.120; 95% confidence interval [CI]: 1.053–1.191). Specifically, COVID-19 survivors with IDD had a significantly increased risk of experiencing abnormal breathing (HR, 1.216; 95% CI: 1.077–1.373), abdominal symptoms (HR, 1.259; 95% CI: 1.128–1.406), fatigue (HR, 1.397; 95% CI: 1.216–1.606), anxiety/depression (HR, 1.157; 95% CI: 1.050–1.274), cognitive symptoms (HR, 1.828; 95% CI: 1.529–2.186), myalgia (HR, 1.325; 95% CI: 1.077–1.631), sleep disturbances (HR, 1.481; 95% CI: 1.148–1.910), and cough (HR, 1.315; 95% CI: 1.146–1.508) compared to the non-IDD group.

**Conclusions:**

Patients with IDD might be associated with a higher risk of post-COVID-19 conditions following SARS-CoV-2 infection compared to the general population.

**Supplementary Information:**

The online version contains supplementary material available at 10.1186/s12916-023-03216-8.

## Background

The coronavirus disease 2019 (COVID-19) pandemic has had a devastating impact on individuals with intellectual disability disorder (IDD), a medical condition characterized by significant impairments in intellectual and adaptive functioning. IDD typically begins in childhood and results in lifelong impairments in mobility, language, learning, self-care, and independent living. Studies conducted in the early pandemic period revealed that patients with intellectual disability, cerebral palsy, and Down syndrome, all of whom are classified as IDD, are more susceptible to contracting severe acute respiratory syndrome coronavirus 2 (SARS-CoV-2) and experiencing severe consequences of COVID-19 [1–3]. The Centers for Disease Control and Prevention report that evidence from multiple countries since May 2021 indicates that people with IDDs face a higher risk of contracting and dying from COVID-19 compared to the general population [2]. A study involving New York State residents found that those with IDD had a mortality rate nearly eight times higher than that of the general population, highlighting the severity of the risk they face [4].

Comorbidities in patients often contribute to a heightened risk of post-COVID-19 conditions [5], a risk further amplified in patients with IDD due to their higher prevalence of comorbidities [6]. Their heightened susceptibility is not just due to biological factors but also stems from the interplay of social determinants and healthcare disparities they face [7, 8]. Despite the burgeoning research on COVID-19's long-term impacts, its specific effects on individuals with IDD, particularly in relation to post-COVID-19 conditions, remain notably understudied. This research gap, which has been highlighted in several studies [9, 10], underscores an urgent need for data - a need our research seeks to address. It is crucial to investigate the prevalence of post-COVID-19 conditions within this population to gain insight into how post-COVID-19 conditions affect patients with IDD can aid future social care planning and management improvement. To date, no study has specifically probed post-COVID-19 conditions in patients with IDD. Consequently, this study aims to utilize the extensive global TriNetX research platform to evaluate the relative prevalence of post-COVID-19 conditions on patients with IDD versus the general population.

## Methods

### Data source

This study used data from the TriNetX Research Network, a collaborative health research platform that aggregates de-identified patient-level data from electronic health records, including demographic data, diagnoses, procedures, medications, laboratory data, genomic data, and types of healthcare organization (HCO) visits. TriNetX contains data from over 120 HCOs globally, typically academic health centers that collect data from their affiliated facilities, including main and satellite hospitals and outpatient clinics. For the present analysis, we used the Research Network, which contains the data of over 107 million patients from 76 HCOs. The TriNetX platform includes built-in tools for analyzing patient-level data, and the results are provided to researchers in an aggregate form. Detailed information on the database can be found online [11]. Written informed consent was not required because TriNetX contains anonymized data. The Institutional Review Board of the Chi Mei Medical Center approved the study protocol (no. 11202-002).

### Patient selection

We compared the risk of post-COVID-19 conditions between patients with and without an IDD. The IDD group comprised patients aged ≥ 18 years with a diagnosis of IDD before testing positive for SARS-CoV-2 or receiving a COVID-19 diagnosis (Table S1). We created exclusive categories for patients with commonly reported IDDs using International Classification of Diseases (ICD-10) codes: intellectual disability, ICD-10 F70-79; Down syndrome, ICD-10 Q90.9; and cerebral palsy, ICD-10 G80, as previous described [1, 12–15].

The non-IDD group was identified using identical criteria but without any IDD diagnosis (Table S2). To ensure a 180-day follow-up for each patient, at least two medical encounters with HCOs were required between March 1, 2020, and October 1, 2022. Patients diagnosed with post-COVID-19 conditions within 1 year before the index date or those requiring initial hospitalization were excluded Index date was defined as the date of diagnosing COVID-19 and only first episode of SARS-CoV-2 infection was included (Table S3).

### Primary outcome

The primary outcome of this study was a composite outcome consisting of 12 clinical features of post-COVID-19 conditions observed 90–180 days after the index event. The follow up period was used based on the definition of post-COVID-19 conditions by World Health Organization – the symptoms persist for 3 months after the initial infection. These features include chest/throat pain, abnormal breathing, abdominal symptoms, fatigue/malaise, anxiety/depression, headache, cognitive dysfunction, myalgia, loss of taste or smell, sleep disturbance, cough, and palpitations [16–18] and were identified using ICD-10 code (Table S4). In addition to these clinical features, survival and time-to-event outcomes following the index event were also evaluated using Kaplan-Meier and log rank testing to provide further insights into the potential progression and duration of these post-COVID-19 conditions in individuals with IDD versus the general population.

### Secondary outcomes

The secondary outcomes encompassed the components of the primary outcome, specifically post-acute COVID-19 conditions, such as chest/throat pain, abnormal breathing, abdominal symptoms, fatigue/malaise, anxiety/depression, headache, cognitive dysfunction, myalgia, loss of taste or smell, sleep disturbance, cough, and palpitations between 90 and 180 days after the index date.

### Covariates

We considered 39 variables to adjust for imbalances in baseline characteristics between the IDD and non-IDD groups. We utilized a list of both confirmed and suspected risk factors for COVID-19 and more severe cases of the illness, which included demographics (such as age, sex, and ethnicity), adverse socioeconomic determinants of health (including “problems related to education and literacy,” “problems related to employment and unemployment,” and “problems related to housing and economic circumstances,” as defined by ICD-10), and comorbidities (such as obesity, hypertension, diabetes mellitus, chronic kidney disease [CKD], asthma, chronic lower respiratory diseases, ischemic heart disease, neoplasm, chronic liver diseases, stroke, dementia, rheumatoid arthritis, lupus, psoriasis, human immunodeficiency virus [HIV] infection, mood disorders, and psychotic disorders) [19–21]. We compiled all baseline characteristics and underlying conditions using the most recent data within the three years before the index date. If multiple data points were available within this period, we chose the one closest to the index date.

### Statistical analysis

We used the built-in propensity score-matching function of the TriNetX platform to ensure a 1:1 match between the participants in the IDD and non-IDD groups. This was enabled by employing a nearest-neighbor greedy matching algorithm with a caliper width of 0.1 pooled standard deviation. The propensity score was assigned as the probability of exposure to IDD or non-IDD patients with the covariates included in the regression model and then used to balance the differences between groups. Standardized differences were computed to assess the inequality and the confounding effect between groups. Any differences in absolute values < 0.1 indicated a good match between groups [22]. To evaluate the survival and the time-to-event data, we employed the Kaplan-Meier survival analysis. The differences between survival curves were analyzed using the log-rank test. We used Cox proportional hazard models to calculate the hazard ratios, which involved adjusting for potential confounding variables. The hazard ratios, with corresponding confidence intervals, were derived to examine the relative risk of post-COVID-19 conditions in the IDD population compared to the control group. All tests were two-sided, and *p*-values less than 0.05 were considered statistically significant.

For the subgroup analysis, we compared the risks of post-COVID-19 conditions between IDD and non-IDD groups. This comparison was stratified by age (18–64 and ≥ 65 years), sex, vaccine status (unvaccinated or vaccinated with at least one dose 14 days before the SARS-CoV-2 infection), and race (white and non-white).

## Results

### Patient characteristics

**Fig. 1:**
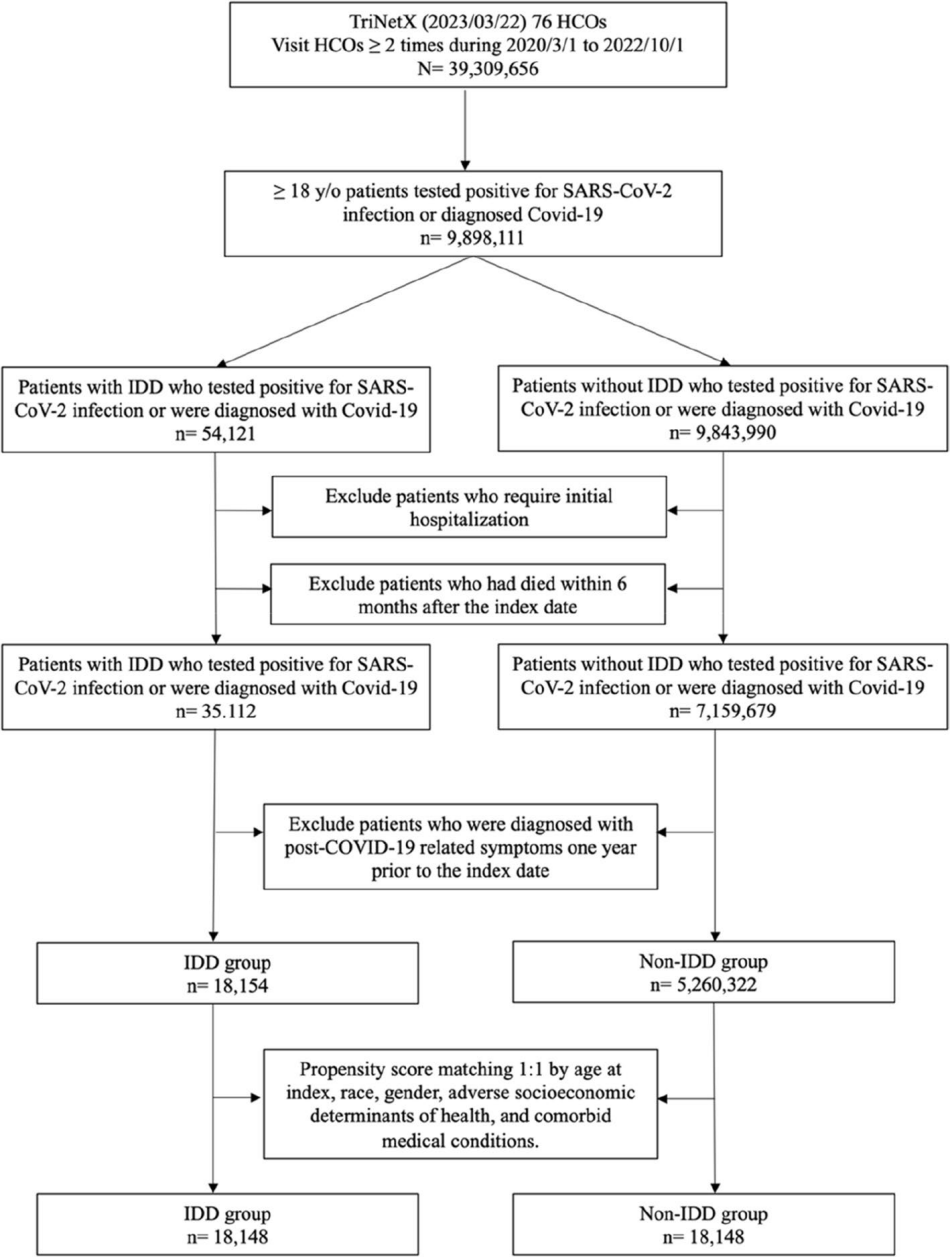
Flowchart of patient selection and cohort construction of the cohort

Between March 1, 2020, and October 1, 2022, 18,154 patients with IDD were identified, and 5,260,322 patients without IDD were subjected to matching (Fig. 1). Table 1 summarizes the baseline characteristics of the IDD and non-IDD groups, before and after propensity score matching. Before matching, the IDD and non-IDD groups showed significant differences. The IDD group was younger compared to the non-IDD group (38.9 ± 17.0 vs. 46.3 ± 18.5 years) and had a higher proportion of white individuals (67.19% vs. 51.07%). Additionally, the IDD group exhibited a higher prevalence of unfavorable socioeconomic determinants of health compared to the non-IDD group, including issues related to housing and economic circumstances (4.02% vs. 0.35%), employment and unemployment (1.28% vs. 0.13%). Moreover, the IDD group had a higher prevalence of underlying conditions, with the top three being hypertensive diseases (21.3% vs. 12.39%), overweight and obesity (16.31% vs. 5.49%), and mood disorders (15.35% vs. 3.16%). After propensity score matching, the absolute standardized difference between the IDD and non-IDD groups was < 0.1, indicating a high level of balance between the two groups [22].

**Table 1:** Comparison of characteristics of patients with intellectual disability disorders (IDDs) and non-IDDs group before and after matching

	Before matching			After matching		
	IDD group (*n* = 18,154)	Non-IDD group (*n* = 5,260,322)	Std diff	IDD group (*n* = 18,148)	Non-IDD group (*n* = 18,148)	Std diff
**Age at Index**	38.9 ± 17.0	46.3 ± 18.5	0.419	38.9 ± 17.0	39.8 ± 17.5	0.053
**Sex**						
Female	9207 (50.72%)	2918736 (55.49%)	0.096	9204 (50.72%)	8854 (48.79%)	0.039
Male	8945 (49.27%)	2338751 (44.46%)	0.097	8942 (49.27%)	9285 (51.16%)	0.038
**Race**						
White	12198 (67.19%)	2686343 (51.07%)	0.332	12194 (67.19%)	12377 (68.2%)	0.022
Black or African American	3591 (19.78%)	686954 (13.06%)	0.182	3589 (19.78%)	3656 (20.15%)	0.009
Asian	344 (1.9%)	111763 (2.13%)	0.016	344 (1.9%)	200 (1.1%)	0.065
Unknown Race	1933 (10.65%)	1753455 (33.33%)	0.569	1933 (10.65%)	1880 (10.36%)	0.010
**Socioeconomic determinants of health**						
Problems related to education and literacy	79 (0.44%)	2593 (0.05%)	0.079	78 (0.43%)	64 (0.35%)	0.012
Problems related to employment and unemployment	233 (1.28%)	6852 (0.13%)	0.138	231 (1.27%)	188 (1.04%)	0.022
Problems related to housing and economic circumstances	729 (4.02%)	18641 (0.35%)	0.252	727 (4.01%)	752 (4.14%)	0.007
**Comorbidities**						
Hypertensive diseases	3835 (21.13%)	651949 (12.39%)	0.235	3831 (21.11%)	4021 (22.16%)	0.025
Overweight and obesity	2961 (16.31%)	288719 (5.49%)	0.353	2956 (16.29%)	2972 (16.38%)	0.002
Type 2 diabetes mellitus	2390 (13.17%)	271320 (5.16%)	0.280	2385 (13.14%)	2407 (13.26%)	0.004
Ischemic heart diseases	687 (3.78%)	158517 (3.01%)	0.043	682 (3.76%)	970 (5.35%)	0.076
Cerebral infarction	765 (4.21%)	45595 (0.87%)	0.214	760 (4.19%)	703 (3.87%)	0.016
Neoplasms	2346 (12.92%)	438104 (8.33%)	0.150	2342 (12.91%)	2282 (12.57%)	0.010
Nicotine dependence	1723 (9.49%)	205371 (3.9%)	0.225	1719 (9.47%)	1659 (9.14%)	0.011
Mood disorders	2787 (15.35%)	166147 (3.16%)	0.430	2781 (15.32%)	2963 (16.33%)	0.027
Schizophrenia and other non-mood psychotic disorders	1331 (7.33%)	23571 (0.45%)	0.362	1325 (7.3%)	1352 (7.45%)	0.006
Mental and behavioral disorders due to psychoactive substance use	2344 (12.91%)	280762 (5.34%)	0.265	2338 (12.88%)	2299 (12.67%)	0.006
Chronic kidney disease						
Chronic kidney disease	912 (5.02%)	114238 (2.17%)	0.154	912 (5.03%)	1016 (5.6%)	0.026
Hypertensive chronic kidney disease	258 (1.42%)	35244 (0.67%)	0.074	258 (1.42%)	273 (1.5%)	0.007
Chronic liver diseases						
Fatty liver	301 (1.66%)	42123 (0.8%)	0.078	301 (1.66%)	353 (1.95%)	0.022
Fibrosis and cirrhosis of liver	150 (0.83%)	18692 (0.36%)	0.061	149 (0.82%)	151 (0.83%)	0.001
Alcoholic liver disease	17 (0.09%)	7917 (0.15%)	0.016	17 (0.09%)	66 (0.36%)	0.057
Chronic hepatitis	12 (0.07%)	1307 (0.03%)	0.019	12 (0.07%)	15 (0.08%)	0.006
Portal hypertension	31 (0.17%)	6199 (0.12%)	0.014	31 (0.17%)	47 (0.26%)	0.019
Chronic lower respiratory diseases						
Chronic obstructive pulmonary disease	361 (1.99%)	65012 (1.24%)	0.060	361 (1.99%)	489 (2.7%)	0.047
Asthma	1870 (10.3%)	166430 (3.16%)	0.288	1865 (10.28%)	1885 (10.39%)	0.004
Bronchitis	299 (1.65%)	42117 (0.8%)	0.077	299 (1.65%)	359 (1.98%)	0.025
Simple and mucopurulent chronic bronchitis	25 (0.14%)	3998 (0.08%)	0.019	25 (0.14%)	19 (0.11%)	0.010
Emphysema	87 (0.48%)	21164 (0.4%)	0.012	84 (0.46%)	122 (0.67%)	0.028
Dementia						
Vascular dementia	20 (0.11%)	1536 (0.03%)	0.031	20 (0.11%)	15 (0.08%)	0.009
Alzheimer’s disease	39 (0.22%)	2903 (0.06%)	0.043	38 (0.21%)	26 (0.14%)	0.016
Immune disorders						
Human immunodeficiency virus disease	506 (2.79%)	17105 (0.33%)	0.200	502 (2.77%)	395 (2.18%)	0.038
Sarcoidosis	24 (0.13%)	6144 (0.12%)	0.004	23 (0.13%)	33 (0.18%)	0.014
Immunodeficiency with predominantly antibody defects	37 (0.2%)	2631 (0.05%)	0.043	37 (0.2%)	16 (0.09%)	0.030
Psoriasis	211 (1.16%)	25074 (0.48%)	0.076	208 (1.15%)	174 (0.96%)	0.018
Rheumatoid arthritis	134 (0.74%)	24663 (0.47%)	0.035	132 (0.73%)	153 (0.84%)	0.013
Systemic lupus erythematosus	44 (0.24%)	8076 (0.15%)	0.020	43 (0.24%)	73 (0.4%)	0.029

### Primary outcome

**Fig. 2:**
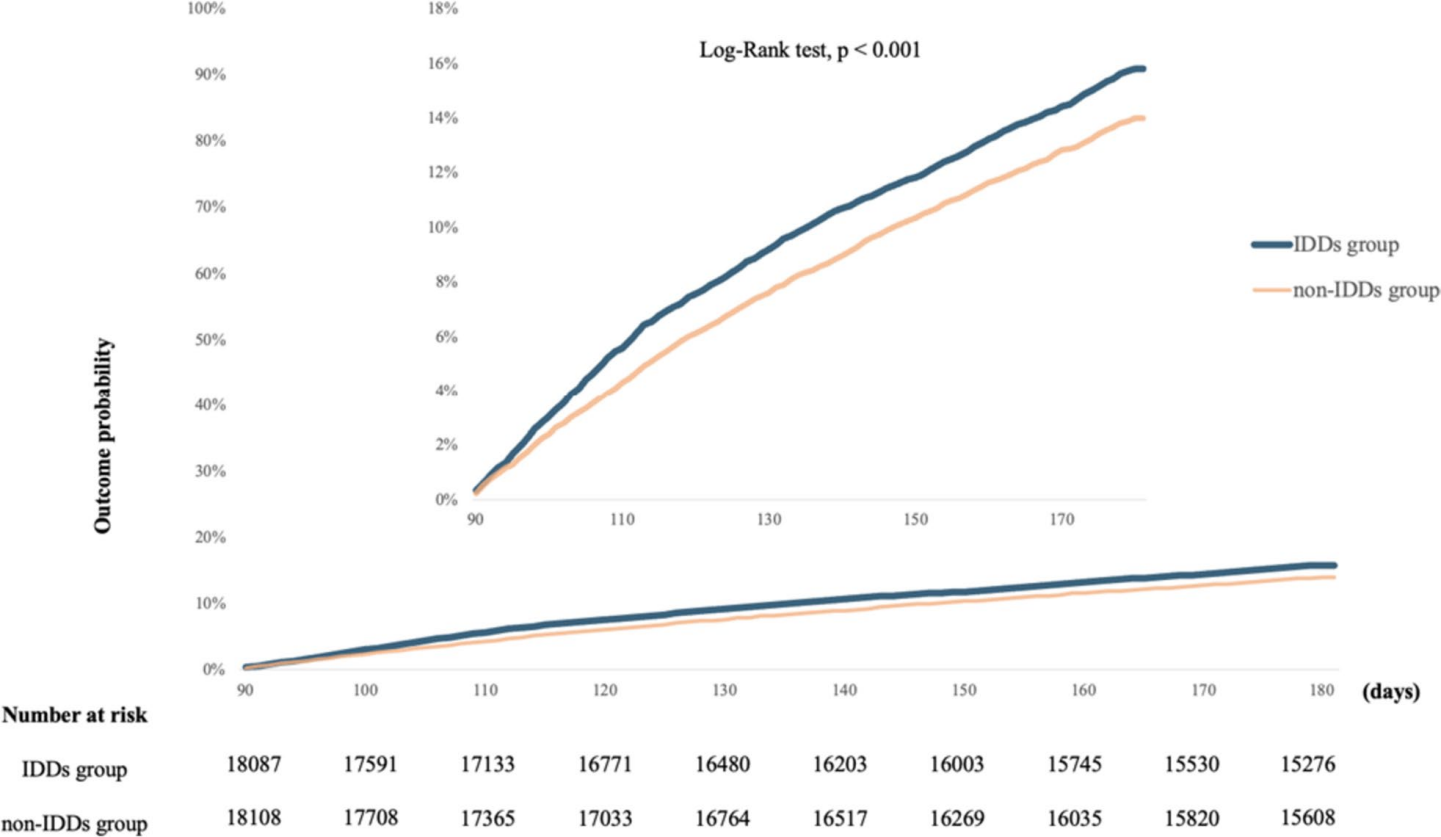
The probability of the primary outcome—a composite of any post-COVID-19 conditions This figure incorporates two Kaplan-Meier curves with different scales. The lower curve displays the range from 0 to 100%, while the upper curve provides a magnified view from 0 to 18% for more detailed observation of variations. The blue curve represents IDDs group, and the orange curve indicates non-IDDs group

During the 90–180 day follow-up, post-COVID-19 conditions were experienced by 15.82% of patients with IDD (95% CI: 15.22%-16.45%). In contrast, 13.99% of patients without IDD experienced post-COVID-19 conditions (95% CI: 13.39%-14.61%). The primary composite outcome for any post-COVID-19 condition was significantly higher between 90 and 180 days in the IDD group than in the control group (HR, 1.120; 95% CI: 1.053–1.191; Fig. 2).

### Secondary outcomes

The most prevalent post-COVID-19 condition in the IDD group was anxiety/depression (6.59%, 95% CI: 6.19–7.02), followed by abdominal symptoms (5.42%, 95% CI: 5.06–5.82), abnormal breathing (4.41%, 95% CI: 4.08–4.77), fatigue (3.62%, 95% CI: 3.32–3.95), and cough (3.59%, 95% CI: 3.29–3.91) (Table 2). Moreover, the IDD group exhibited a significantly increased risk of experiencing abnormal breathing (HR, 1.216; 95% CI: 1.077–1.373), abdominal symptoms (HR, 1.259; 95% CI: 1.128–1.406), fatigue (HR, 1.397; 95% CI: 1.216–1.606), anxiety/depression (HR, 1.157; 95% CI: 1.050–1.274), cognitive symptoms (HR, 1.828; 95% CI: 1.529–2.186), myalgia (HR, 1.325; 95% CI: 1.077–1.631), sleep disturbance (HR, 1.481; 95% CI: 1.148–1.910), and cough (HR, 1.315; 95% CI: 1.146–1.508) compared to the non-IDD group (Table 2).

**Table 2:** The hazard ratio and incidence for comparing matched intellectual disability disorder (IDD) and non-IDD group for the primary composite outcome and its constituents

Outcome	Incidence of post-COVID-19 condition (%)		Hazard ratio	(95%CI)	*P* value*
	IDD group	Non-IDD group			
Chest/throat pain:	3.21%	2.73%	1.148	(0.998, 1.320)	0.053
Abnormal breathing	4.41%	3.33%	1.216	(1.077, 1.373)	**0.002**
Abdominal symptoms	5.42%	4.06%	1.259	(1.128, 1.406)	** < .0001**
Fatigue:	3.62%	2.50%	1.397	(1.216, 1.606)	** < .0001**
Anxiety/Depression	6.59%	5.53%	1.157	(1.050, 1.274)	**0.003**
Headache	3.39%	2.57%	1.260	(1.097, 1.448)	** < .0001**
Cognitive symptoms	2.61%	1.28%	1.828	(1.529, 2.186)	** < .0001**
Myalgia	1.58%	0.98%	1.325	(1.077, 1.631)	**0.008**
Loss of taste/smell	0.01%	0.02%	0.304	(0.061, 1.506)	0.122
Sleep disturbance	1.11%	0.74%	1.481	(1.148, 1.910)	**0.002**
Cough	3.59%	2.54%	1.315	(1.146, 1.508)	** < .0001**
Palpitation	1.36%	1.29%	1.104	(0.893, 1.365)	0.362
Any post-COVID-19 condition	15.82%	13.99%	1.120	(1.053, 1.191)	** < .0001**

^*^bold form indicated *p* < 0.05

### Subgroup analysis

**Fig. 3:**
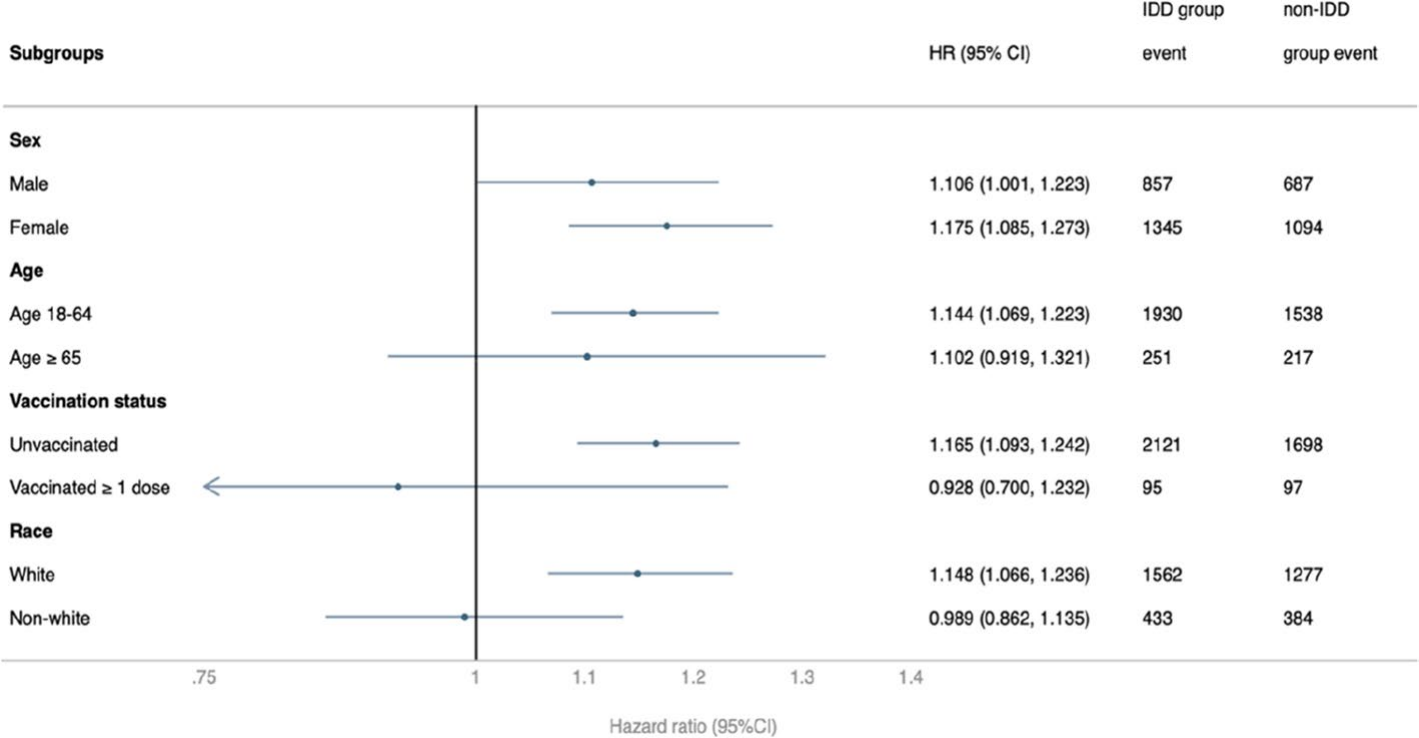
Subgroup analysis of the risk of composite outcome of any post-COVID-19 condition between the intellectual disability disorder (IDD) group and the non-IDD group

The risk of post-COVID-19 conditions in the subgroups based on sex, age, race, and vaccination status was examined (Fig. 3). Compared to the non-IDD group, the IDD group had significantly and consistently higher HR for the primary composite outcome in most subgroups, including male (HR, 1.106; 95% CI: 1.001–1.223), female (HR, 1.175; 95% CI: 1.085–1.273), 18–64-year-old (HR, 1.144; 95% CI: 1.069–1.223), unvaccinated (HR, 1.165; 95% CI: 1.093–1.242), and white (HR, 1.148; 95% CI: 1.066–1.236). When analyzing individual outcomes across various subgroups (Figure S1-S4), the results showed variations among different subgroups, and this variability could be the relatively limited sample sizes within each of these divided subgroups.

## Discussion

In the present study, we evaluated post-COVID-19 conditions and determined their relative impact on patients with IDD compared to the general population. The study included 36,308 patients with COVID-19 and produced several significant findings. Patients with IDD had a significantly higher risk of the composite outcomes of any post-COVID-19 condition over a period of 90–180 days after SARS-CoV-2 infection compared to the non-IDD population. Furthermore, patients with IDD had the higher risk of the following nine conditions included abnormal breathing, abdominal symptoms, fatigue, anxiety/depression, cognitive symptoms, myalgia, sleep disturbances, and coughing. The higher risk of the composite outcomes of any post-COVID-19 conditions in the IDD group was consistent in the subgroup analyses by female sex, age group of 18–64 years, unvaccinated patients, and white race. Therefore, clinicians should be aware of post-COVID-19 conditions for patients with IDD who have survived COVID-19. Recognition of post-COVID-19 conditions requires clinicians and caregivers to look for subtle changes in behavior and functional requirements. Vulnerable individuals should not be excluded in response to the pandemic.

In concordance with existing literature, our findings indicate a significantly higher prevalence of comorbid conditions in the IDD population, including hypertensive and pulmonary diseases, obesity, and metabolic and mental health disorders [23, 24] Particularly, the association between obesity and increased COVID-19 severity warrants focused attention [25]. Our data align with studies by Courtenay et al. and Gleason et al., which document the vulnerability of the IDD population to both COVID-19 and its post-infection sequelae, possibly due to a confluence of intellectual disability and prevalent comorbidities [26, 27]. Further research is needed to elucidate any direct causal relationships.

This was the first study to provide evidence on the prevalence of post-COVID-19 conditions in patients with an IDD. Patients with IDD are at a higher risk of experiencing post-COVID-19 conditions after SARS-CoV-2 infection compared to the general population. Raveendran et al.’s review of post-COVID-19 conditions identified the potential pathophysiological mechanisms associated with post-COVID-19 conditions, including organ damage, inflammation, immune responses, comorbidity interactions, psychological factors, deconditioning, and social and financial impacts [28], which are also relevant to patients with IDD. Additionally, socioeconomic factors and obstacles to receiving appropriate healthcare may contribute to an increased risk of post-COVID-19 conditions in this population [7]. Evidence collected from the United Kingdom suggests that post-COVID-19 conditions are more common in those with activity-limiting health conditions or disabilities and in those living in more deprived areas, which are common characteristics of patients with IDD [8]. As a result, patients with IDD are more susceptible to post-COVID-19 conditions owing to a combination of factors, including negative socioeconomic health determinants, the presence of potential comorbidities, and difficulties in obtaining suitable healthcare.

Subgroup analyses suggests that having the COVID-19 vaccine might be beneficial in preventing composite outcomes of any post-COVID conditions. Patients who received at least one dose of the vaccine showed no significant difference in the occurrence of post-COVID conditions compared to the general population. These findings are consistent with those of Taquet et al., who demonstrated that the administration of at least two doses of the vaccine was linked with a reduced risk of a broad range of sequelae associated with SARS-CoV-2 infections [18]. Additionally, a systematic review conducted by Notarte et al. revealed that vaccination prior to infection could mitigate the risk of developing subsequent post-COVID-19 conditions [29].

The present study had several strengths. First, we used a large population-based and dynamically maintained database that allowed us to investigate a broader global population from four countries - Brazil, Taiwan, the United States, and Poland and examine more recent timeframes. Second, we employed a rigorous methodology to address the challenge of determining whether the diagnosis of post-COVID-19 conditions was related to pre-existing underlying conditions or COVID-19. To this end, we excluded patients with a history of post-COVID-19 symptoms before SARS-CoV-2 infection. This approach confirmed that post-COVID-19 symptoms occur after SARS-CoV-2 infection and reduced the likelihood of old symptoms being mistaken for post-COVID-19 conditions. We carefully matched the groups in terms of baseline characteristics, negative socioeconomic determinants of health, and pre-existing medical comorbidities. This approach helped avoid bias caused by these confounding factors and strengthened the internal validity of our findings. Finally, this study is the first to provide evidence of the prevalence of post-COVID-19 conditions in patients with an IDD. Our research provides new insight on the sustained vulnerability of the IDD population beyond the acute stage of COVID-19. Additionally, there's a higher likelihood of IDD patients developing post-COVID-19 conditions compared to the general population. Moreover, the observed correlations may have several implications for clinical practice and policy. For healthcare professionals, these findings highlight the importance of closely monitoring patients with IDD who have recovered from COVID-19 for the development of long-term sequelae. For policymakers, the results underscore the necessity of creating health strategies that proactively address the unique needs of the IDD population during pandemics. We also suggest developing interventions specifically for individuals with IDD who have had COVID-19, as they might have unmet healthcare needs.

This study has several limitations. First, the data used were subject to inherent biases because of the use of registry databases, including misdiagnosis, inaccurate coding, and documentation errors. Second, recognizing post-COVID-19 conditions in patients with an IDD can be challenging because of communication difficulties and poor intellectual functioning. Therefore, this cohort may have a limited ability to articulate symptoms or distress, potentially leading to an underestimation of the prevalence of post-COVID-19 conditions. Third, although we performed propensity score matching to minimize confounding factors, residual confounding factors could not entirely be eliminated. The present analysis lacked information on potentially relevant factors, such as socioeconomic factors and social support. Fourth, while our data indicate associations, they do not directly prove a causal relationship. Despite our rigorous methodology wherein we excluded patients with a history of post-COVID-19-related conditions, aiming to ensure that the symptoms we observed had manifested after the COVID-19 infection, establishing causality remains intricate. Lastly, given that the sample includes four countries, there could be some factors, such as the differences in healthcare and welfare systems, as well as variations in the prevalence of COVID-19 and post-COVID conditions, that might potentially influence the relationship between IDD and post-COVID conditions at the country level.

## Conclusions

Compared to the general population, patients with IDD might be associated with a higher risk of post-COVID-19 conditions after SARS-CoV-2 infection. Caregivers and clinicians should identify and manage these potential conditions to provide proper care and support.

### Supplementary Information

**Additional file 1: Table S1.** Query Criteria for Cohort (query name: IDD). **Table S2.** Query Criteria for Cohort (query name: W/O IDD). **Table S3.** Index Events Used in this Analysis. **Table S4.** Outcome Definitions. **Fig. S1.** Forest plots of primary outcome and its components stratified by sex. **Fig. S2.** Forest plots of primary outcome and its components stratified by age. **Fig. S3.** Forest plots of primary outcome and its components stratified by vaccine status. **Fig. S4.** Forest plots of primary outcome and its components stratified by race.

## Data Availability

All data generated or analyzed during this study are included in this published article and will be available upon request to CCL.

## Abbreviations

COVID-19: Coronavirus disease 2019
CI: Confidence interval
ED: Emergency department
EHR: Electronic health record
HCO: Healthcare organization
HR: Hazard ratio
IDD: Intellectual disability disorder
PSM: Propensity score-matching
SARS-CoV-2: Severe acute respiratory syndrome coronavirus 2
