# Supplementary material for: Post-COVID-19 condition risk in patients with intellectual and developmental disabilities: a retrospective cohort study involving 36,308 patients
Source: BMC Med. 2023 Dec 20;21:505. doi: 10.1186/s12916-023-03216-8 (PMC10731815; doi:10.1186/s12916-023-03216-8)

**Supplementary methods**

# Introduction

TriNetX is the global federated health research network providing access to electronic medical records (diagnoses, procedures, medications, laboratory values, genomic information) across large healthcare organizations (HCOs). This report was run on the set of HCOs grouped into a network called Research. This network included 76 HCO(s).

# Methods

The analysis process includes two main steps: 1) Defining the cohorts through query criteria; 2) Setting up and running the analysis. Setting up the analysis requires definitions for the index event, outcomes criteria, and the time frame. Compare outcomes supports four analyses: Measures of Association, Survival, Number of Instances and Lab result distribution. These analyses have additional options that are listed in the Outcomes Definitions and Analyses Specifications section below. Furthermore, characteristics of the cohorts that are balanced using propensity score matching are also included in the Propensity Score Matching section.

## Cohorts definition

This section lists all terms used in the definitions of the two cohorts.

**Table S1:** Query Criteria for Cohort (query name: IDD)

This query was run on the network Research with 76 HCO(s) queried and 76 HCO(s) responded. A total of 59 provider(s) responded with patients.

|  | | | | | |
| --- | --- | --- | --- | --- | --- |
| Ungrouped terms | | | | | |
|  | must have |  | demographics | Age | Age (at least 18 years) |
| Group 1 | | | | | |
|  | **visit** | | | | |
|  | must have |  | visit | TNX:Visit | Visit |
|  | number of occurrences | | Greater than or equal to 2 instances | | |
|  | date constraint | | The terms in this group occurred between Mar 1, 2020 and Oct 1, 2022 | | |
| Group 2 | | | | | |
|  | **Group 2A Covid-19** | | | | |
|  | must have | any of | diagnosis | UMLS:ICD10CM:U07.1 | COVID-19 |
|  |  |  | diagnosis | UMLS:ICD10CM:J12.81 | Pneumonia due to SARS-associated coronavirus |
|  |  |  | diagnosis | UMLS:ICD10CM:J12.82 | Pneumonia due to coronavirus disease 2019 |
|  |  |  | laboratory | TNX:9088 | SARS coronavirus 2 and related RNA [Presence] |
|  |  |  | laboratory | UMLS:LNC:94309-2 | SARS-CoV-2 (COVID-19) RNA [Presence] in Specimen by NAA with probe detection |
|  |  |  | laboratory | UMLS:LNC:94500-6 | SARS-CoV-2 (COVID-19) RNA [Presence] in Respiratory specimen by NAA with probe detection |
|  |  |  | laboratory | UMLS:LNC:94502-2 | SARS-related coronavirus RNA [Presence] in Respiratory specimen by NAA with probe detection |
|  |  |  | laboratory | UMLS:LNC:95406-5 | SARS-CoV-2 (COVID-19) RNA [Presence] in Nose by NAA with probe detection |
|  |  |  | laboratory | UMLS:LNC:94565-9 | SARS-CoV-2 (COVID-19) RNA [Presence] in Nasopharynx by NAA with non-probe detection |
|  |  |  | laboratory | UMLS:LNC:95608-6 | SARS-CoV-2 (COVID-19) RNA [Presence] in Respiratory specimen by NAA with non-probe detection |
|  |  |  | laboratory | UMLS:LNC:94759-8 | SARS-CoV-2 (COVID-19) RNA [Presence] in Nasopharynx by NAA with probe detection |
|  |  |  | laboratory | UMLS:LNC:94845-5 | SARS-CoV-2 (COVID-19) RNA [Presence] in Saliva (oral fluid) by NAA with probe detection |
|  |  |  | laboratory | UMLS:LNC:96119-3 | SARS-CoV-2 (COVID-19) Ag [Presence] in Upper respiratory specimen by Immunoassay |
|  |  |  | laboratory | UMLS:LNC:94558-4 | SARS-CoV-2 (COVID-19) Ag [Presence] in Respiratory specimen by Rapid immunoassay |
|  | date constraint | | The terms in this group occurred at any time | | |
|  | event relationship | | Any instance of idd occurred on or before the first instance of Covid-19 | | |
|  | **Group 2B idd** | | | | |
|  | must have | any of | diagnosis | UMLS:ICD10CM:G80 | Cerebral palsy |
|  |  |  | diagnosis | UMLS:ICD10CM:Q90.9 | Down syndrome, unspecified |
|  |  |  | diagnosis | UMLS:ICD10CM:F70-F79 | Intellectual Disabilities |
| Group 3 | | | | | |
|  | **Group 3A Covid-19** | | | | |
|  | must have | any of | diagnosis | UMLS:ICD10CM:U07.1 | COVID-19 |
|  |  |  | diagnosis | UMLS:ICD10CM:J12.81 | Pneumonia due to SARS-associated coronavirus |
|  |  |  | diagnosis | UMLS:ICD10CM:J12.82 | Pneumonia due to coronavirus disease 2019 |
|  |  |  | laboratory | TNX:9088 | SARS coronavirus 2 and related RNA [Presence] |
|  |  |  | laboratory | UMLS:LNC:94309-2 | SARS-CoV-2 (COVID-19) RNA [Presence] in Specimen by NAA with probe detection |
|  |  |  | laboratory | UMLS:LNC:94500-6 | SARS-CoV-2 (COVID-19) RNA [Presence] in Respiratory specimen by NAA with probe detection |
|  |  |  | laboratory | UMLS:LNC:94502-2 | SARS-related coronavirus RNA [Presence] in Respiratory specimen by NAA with probe detection |
|  |  |  | laboratory | UMLS:LNC:95406-5 | SARS-CoV-2 (COVID-19) RNA [Presence] in Nose by NAA with probe detection |
|  |  |  | laboratory | UMLS:LNC:94565-9 | SARS-CoV-2 (COVID-19) RNA [Presence] in Nasopharynx by NAA with non-probe detection |
|  |  |  | laboratory | UMLS:LNC:95608-6 | SARS-CoV-2 (COVID-19) RNA [Presence] in Respiratory specimen by NAA with non-probe detection |
|  |  |  | laboratory | UMLS:LNC:94759-8 | SARS-CoV-2 (COVID-19) RNA [Presence] in Nasopharynx by NAA with probe detection |
|  |  |  | laboratory | UMLS:LNC:94845-5 | SARS-CoV-2 (COVID-19) RNA [Presence] in Saliva (oral fluid) by NAA with probe detection |
|  |  |  | laboratory | UMLS:LNC:96119-3 | SARS-CoV-2 (COVID-19) Ag [Presence] in Upper respiratory specimen by Immunoassay |
|  |  |  | laboratory | UMLS:LNC:94558-4 | SARS-CoV-2 (COVID-19) Ag [Presence] in Respiratory specimen by Rapid immunoassay |
|  | date constraint | | The terms in this group occurred at any time | | |
|  | event relationship | | Any instance of Patients requiring initial hospitalization occurred within 1 month on or after the first instance of Covid-19 | | |
|  | **Group 3B Patients requiring initial hospitalization** | | | | |
|  | cannot have |  | visit | UMLS:HL7V3.0:VisitType:IMP | Visit: Inpatient Encounter |
|  |  | or | visit | UMLS:HL7V3.0:VisitType:NONAC | Visit: Inpatient Non-acute |
|  |  | or | visit | UMLS:HL7V3.0:VisitType:OBSENC | Visit: Observation Encounter |
|  |  | or | visit | UMLS:HL7V3.0:VisitType:SS | Visit: Short Stay |
| Group 4 | | | | | |
|  | **Group 4A Covid-19** | | | | |
|  | must have | any of | diagnosis | UMLS:ICD10CM:U07.1 | COVID-19 |
|  |  |  | diagnosis | UMLS:ICD10CM:J12.81 | Pneumonia due to SARS-associated coronavirus |
|  |  |  | diagnosis | UMLS:ICD10CM:J12.82 | Pneumonia due to coronavirus disease 2019 |
|  |  |  | laboratory | TNX:9088 | SARS coronavirus 2 and related RNA [Presence] |
|  |  |  | laboratory | UMLS:LNC:94309-2 | SARS-CoV-2 (COVID-19) RNA [Presence] in Specimen by NAA with probe detection |
|  |  |  | laboratory | UMLS:LNC:94500-6 | SARS-CoV-2 (COVID-19) RNA [Presence] in Respiratory specimen by NAA with probe detection |
|  |  |  | laboratory | UMLS:LNC:94502-2 | SARS-related coronavirus RNA [Presence] in Respiratory specimen by NAA with probe detection |
|  |  |  | laboratory | UMLS:LNC:95406-5 | SARS-CoV-2 (COVID-19) RNA [Presence] in Nose by NAA with probe detection |
|  |  |  | laboratory | UMLS:LNC:94565-9 | SARS-CoV-2 (COVID-19) RNA [Presence] in Nasopharynx by NAA with non-probe detection |
|  |  |  | laboratory | UMLS:LNC:95608-6 | SARS-CoV-2 (COVID-19) RNA [Presence] in Respiratory specimen by NAA with non-probe detection |
|  |  |  | laboratory | UMLS:LNC:94759-8 | SARS-CoV-2 (COVID-19) RNA [Presence] in Nasopharynx by NAA with probe detection |
|  |  |  | laboratory | UMLS:LNC:94845-5 | SARS-CoV-2 (COVID-19) RNA [Presence] in Saliva (oral fluid) by NAA with probe detection |
|  |  |  | laboratory | UMLS:LNC:96119-3 | SARS-CoV-2 (COVID-19) Ag [Presence] in Upper respiratory specimen by Immunoassay |
|  |  |  | laboratory | UMLS:LNC:94558-4 | SARS-CoV-2 (COVID-19) Ag [Presence] in Respiratory specimen by Rapid immunoassay |
|  | date constraint | | The terms in this group occurred at any time | | |
|  | event relationship | | Any instance of died occurred within 6 months on or after the first instance of Covid-19 | | |
|  | **Group 4B died** | | | | |
|  | cannot have |  | demographics | Deceased | Deceased |
| Group 5 | | | | | |
|  | **Group 5A Covid-19** | | | | |
|  | must have | any of | diagnosis | UMLS:ICD10CM:U07.1 | COVID-19 |
|  |  |  | diagnosis | UMLS:ICD10CM:J12.81 | Pneumonia due to SARS-associated coronavirus |
|  |  |  | diagnosis | UMLS:ICD10CM:J12.82 | Pneumonia due to coronavirus disease 2019 |
|  |  |  | laboratory | TNX:9088 | SARS coronavirus 2 and related RNA [Presence] |
|  |  |  | laboratory | UMLS:LNC:94309-2 | SARS-CoV-2 (COVID-19) RNA [Presence] in Specimen by NAA with probe detection |
|  |  |  | laboratory | UMLS:LNC:94500-6 | SARS-CoV-2 (COVID-19) RNA [Presence] in Respiratory specimen by NAA with probe detection |
|  |  |  | laboratory | UMLS:LNC:94502-2 | SARS-related coronavirus RNA [Presence] in Respiratory specimen by NAA with probe detection |
|  |  |  | laboratory | UMLS:LNC:95406-5 | SARS-CoV-2 (COVID-19) RNA [Presence] in Nose by NAA with probe detection |
|  |  |  | laboratory | UMLS:LNC:94565-9 | SARS-CoV-2 (COVID-19) RNA [Presence] in Nasopharynx by NAA with non-probe detection |
|  |  |  | laboratory | UMLS:LNC:95608-6 | SARS-CoV-2 (COVID-19) RNA [Presence] in Respiratory specimen by NAA with non-probe detection |
|  |  |  | laboratory | UMLS:LNC:94759-8 | SARS-CoV-2 (COVID-19) RNA [Presence] in Nasopharynx by NAA with probe detection |
|  |  |  | laboratory | UMLS:LNC:94845-5 | SARS-CoV-2 (COVID-19) RNA [Presence] in Saliva (oral fluid) by NAA with probe detection |
|  |  |  | laboratory | UMLS:LNC:96119-3 | SARS-CoV-2 (COVID-19) Ag [Presence] in Upper respiratory specimen by Immunoassay |
|  |  |  | laboratory | UMLS:LNC:94558-4 | SARS-CoV-2 (COVID-19) Ag [Presence] in Respiratory specimen by Rapid immunoassay |
|  | date constraint | | The terms in this group occurred at any time | | |
|  | event relationship | | Any instance of long covid occurred within 1 year and 14 days before the first instance of Covid-19 | | |
|  | **Group 5B long covid** | | | | |
|  | cannot have |  | diagnosis | UMLS:ICD10CM:R07 | Pain in throat and chest |
|  |  | or | diagnosis | UMLS:ICD10CM:R06 | Abnormalities of breathing |
|  |  | or | diagnosis | UMLS:ICD10CM:R10 | Abdominal and pelvic pain |
|  |  | or | diagnosis | UMLS:ICD10CM:R19.4 | Change in bowel habit |
|  |  | or | diagnosis | UMLS:ICD10CM:R19.7 | Diarrhea, unspecified |
|  |  | or | diagnosis | UMLS:ICD10CM:G93.3 | Postviral and related fatigue syndromes |
|  |  | or | diagnosis | UMLS:ICD10CM:R53 | Malaise and fatigue |
|  |  | or | diagnosis | UMLS:ICD10CM:F30-F39 | Mood [affective] disorders |
|  |  | or | diagnosis | UMLS:ICD10CM:F40-F48 | Anxiety, dissociative, stress-related, somatoform and other nonpsychotic mental disorders |
|  |  | or | diagnosis | UMLS:ICD10CM:R51 | Headache |
|  |  | or | diagnosis | UMLS:ICD10CM:G43 | Migraine |
|  |  | or | diagnosis | UMLS:ICD10CM:G44 | Other headache syndromes |
|  |  | or | diagnosis | UMLS:ICD10CM:R40 | Somnolence, stupor and coma |
|  |  | or | diagnosis | UMLS:ICD10CM:R41 | Other symptoms and signs involving cognitive functions and awareness |
|  |  | or | diagnosis | UMLS:ICD10CM:R48 | Dyslexia and other symbolic dysfunctions, not elsewhere classified |
|  |  | or | diagnosis | UMLS:ICD10CM:G93.40 | Encephalopathy, unspecified |
|  |  | or | diagnosis | UMLS:ICD10CM:G31.84 | Mild cognitive impairment of uncertain or unknown etiology |
|  |  | or | diagnosis | UMLS:ICD10CM:G30 | Alzheimer's disease |
|  |  | or | diagnosis | UMLS:ICD10CM:G31.0 | Frontotemporal dementia |
|  |  | or | diagnosis | UMLS:ICD10CM:G31.83 | Neurocognitive disorder with Lewy bodies |
|  |  | or | diagnosis | UMLS:ICD10CM:F01 | Vascular dementia |
|  |  | or | diagnosis | UMLS:ICD10CM:F02 | Dementia in other diseases classified elsewhere |
|  |  | or | diagnosis | UMLS:ICD10CM:F03 | Unspecified dementia |
|  |  | or | diagnosis | UMLS:ICD10CM:F05 | Delirium due to known physiological condition |
|  |  | or | diagnosis | UMLS:ICD10CM:F06.8 | Other specified mental disorders due to known physiological condition |
|  |  | or | diagnosis | UMLS:ICD10CM:M79.1 | Myalgia |
|  |  | or | diagnosis | UMLS:ICD10CM:M60 | Myositis |
|  |  | or | diagnosis | UMLS:ICD10CM:R43.8 | Other disturbances of smell and taste |
|  |  | or | diagnosis | UMLS:ICD10CM:G47.0 | Insomnia |
|  |  | or | diagnosis | UMLS:ICD10CM:G47.9 | Sleep disorder, unspecified |
|  |  | or | diagnosis | UMLS:ICD10CM:R05 | Cough |
|  |  | or | diagnosis | UMLS:ICD10CM:R00.2 | Palpitations |
|  |  | or | diagnosis | UMLS:ICD10CM:U09.9 | Post COVID-19 condition, unspecified |

### **Table S2:** Query Criteria for Cohort (query name: W/O IDD)

### This query was run on the network Research with 76 HCO(s) queried and 76 HCO(s) responded. A total of 73 provider(s) responded with patients.

| Ungrouped terms | | | | | |
| --- | --- | --- | --- | --- | --- |
|  | must have |  | demographics | Age | Age (at least 18 years (most recent occurrence)) |
| Group 1 | | | | | |
|  | **visit** | | | | |
|  | must have |  | visit | TNX:Visit | Visit |
|  | number of occurrences | | Greater than or equal to 2 instances | | |
|  | date constraint | | The terms in this group occurred between Mar 1, 2020 and Oct 1, 2022 | | |
| Group 2 | | | | | |
|  | **Group 2A Covid-19** | | | | |
|  | must have | any of | diagnosis | UMLS:ICD10CM:U07.1 | COVID-19 |
|  |  |  | diagnosis | UMLS:ICD10CM:J12.81 | Pneumonia due to SARS-associated coronavirus |
|  |  |  | diagnosis | UMLS:ICD10CM:J12.82 | Pneumonia due to coronavirus disease 2019 |
|  |  |  | laboratory | TNX:9088 | SARS coronavirus 2 and related RNA [Presence] |
|  |  |  | laboratory | UMLS:LNC:94309-2 | SARS-CoV-2 (COVID-19) RNA [Presence] in Specimen by NAA with probe detection |
|  |  |  | laboratory | UMLS:LNC:94500-6 | SARS-CoV-2 (COVID-19) RNA [Presence] in Respiratory specimen by NAA with probe detection |
|  |  |  | laboratory | UMLS:LNC:94502-2 | SARS-related coronavirus RNA [Presence] in Respiratory specimen by NAA with probe detection |
|  |  |  | laboratory | UMLS:LNC:95406-5 | SARS-CoV-2 (COVID-19) RNA [Presence] in Nose by NAA with probe detection |
|  |  |  | laboratory | UMLS:LNC:94565-9 | SARS-CoV-2 (COVID-19) RNA [Presence] in Nasopharynx by NAA with non-probe detection |
|  |  |  | laboratory | UMLS:LNC:95608-6 | SARS-CoV-2 (COVID-19) RNA [Presence] in Respiratory specimen by NAA with non-probe detection |
|  |  |  | laboratory | UMLS:LNC:94759-8 | SARS-CoV-2 (COVID-19) RNA [Presence] in Nasopharynx by NAA with probe detection |
|  |  |  | laboratory | UMLS:LNC:94845-5 | SARS-CoV-2 (COVID-19) RNA [Presence] in Saliva (oral fluid) by NAA with probe detection |
|  |  |  | laboratory | UMLS:LNC:96119-3 | SARS-CoV-2 (COVID-19) Ag [Presence] in Upper respiratory specimen by Immunoassay |
|  |  |  | laboratory | UMLS:LNC:94558-4 | SARS-CoV-2 (COVID-19) Ag [Presence] in Respiratory specimen by Rapid immunoassay |
|  | date constraint | | The terms in this group occurred at any time | | |
|  | event relationship | | Any instance of idd occurred on or before the first instance of Covid-19 | | |
|  | **Group 2B idd** | | | | |
|  | cannot have |  | diagnosis | UMLS:ICD10CM:F70-F79 | Intellectual Disabilities |
|  |  | or | diagnosis | UMLS:ICD10CM:Q90.9 | Down syndrome, unspecified |
|  |  | or | diagnosis | UMLS:ICD10CM:G80 | Cerebral palsy |
| Group 3 | | | | | |
|  | **Group 3A Covid-19** | | | | |
|  | must have | any of | diagnosis | UMLS:ICD10CM:U07.1 | COVID-19 |
|  |  |  | diagnosis | UMLS:ICD10CM:J12.81 | Pneumonia due to SARS-associated coronavirus |
|  |  |  | diagnosis | UMLS:ICD10CM:J12.82 | Pneumonia due to coronavirus disease 2019 |
|  |  |  | laboratory | TNX:9088 | SARS coronavirus 2 and related RNA [Presence] |
|  |  |  | laboratory | UMLS:LNC:94309-2 | SARS-CoV-2 (COVID-19) RNA [Presence] in Specimen by NAA with probe detection |
|  |  |  | laboratory | UMLS:LNC:94500-6 | SARS-CoV-2 (COVID-19) RNA [Presence] in Respiratory specimen by NAA with probe detection |
|  |  |  | laboratory | UMLS:LNC:94502-2 | SARS-related coronavirus RNA [Presence] in Respiratory specimen by NAA with probe detection |
|  |  |  | laboratory | UMLS:LNC:95406-5 | SARS-CoV-2 (COVID-19) RNA [Presence] in Nose by NAA with probe detection |
|  |  |  | laboratory | UMLS:LNC:94565-9 | SARS-CoV-2 (COVID-19) RNA [Presence] in Nasopharynx by NAA with non-probe detection |
|  |  |  | laboratory | UMLS:LNC:95608-6 | SARS-CoV-2 (COVID-19) RNA [Presence] in Respiratory specimen by NAA with non-probe detection |
|  |  |  | laboratory | UMLS:LNC:94759-8 | SARS-CoV-2 (COVID-19) RNA [Presence] in Nasopharynx by NAA with probe detection |
|  |  |  | laboratory | UMLS:LNC:94845-5 | SARS-CoV-2 (COVID-19) RNA [Presence] in Saliva (oral fluid) by NAA with probe detection |
|  |  |  | laboratory | UMLS:LNC:96119-3 | SARS-CoV-2 (COVID-19) Ag [Presence] in Upper respiratory specimen by Immunoassay |
|  |  |  | laboratory | UMLS:LNC:94558-4 | SARS-CoV-2 (COVID-19) Ag [Presence] in Respiratory specimen by Rapid immunoassay |
|  | date constraint | | The terms in this group occurred at any time | | |
|  | event relationship | | Any instance of Patients requiring initial hospitalization occurred within 1 month on or after the first instance of Covid-19 | | |
|  | **Group 3B Patients requiring initial hospitalization** | | | | |
|  | cannot have |  | visit | UMLS:HL7V3.0:VisitType:IMP | Visit: Inpatient Encounter |
|  |  | or | visit | UMLS:HL7V3.0:VisitType:NONAC | Visit: Inpatient Non-acute |
|  |  | or | visit | UMLS:HL7V3.0:VisitType:OBSENC | Visit: Observation Encounter |
|  |  | or | visit | UMLS:HL7V3.0:VisitType:SS | Visit: Short Stay |
| Group 4 | | | | | |
|  | **Group 4A Covid-19** | | | | |
|  | must have | any of | diagnosis | UMLS:ICD10CM:U07.1 | COVID-19 |
|  |  |  | diagnosis | UMLS:ICD10CM:J12.81 | Pneumonia due to SARS-associated coronavirus |
|  |  |  | diagnosis | UMLS:ICD10CM:J12.82 | Pneumonia due to coronavirus disease 2019 |
|  |  |  | laboratory | TNX:9088 | SARS coronavirus 2 and related RNA [Presence] |
|  |  |  | laboratory | UMLS:LNC:94309-2 | SARS-CoV-2 (COVID-19) RNA [Presence] in Specimen by NAA with probe detection |
|  |  |  | laboratory | UMLS:LNC:94500-6 | SARS-CoV-2 (COVID-19) RNA [Presence] in Respiratory specimen by NAA with probe detection |
|  |  |  | laboratory | UMLS:LNC:94502-2 | SARS-related coronavirus RNA [Presence] in Respiratory specimen by NAA with probe detection |
|  |  |  | laboratory | UMLS:LNC:95406-5 | SARS-CoV-2 (COVID-19) RNA [Presence] in Nose by NAA with probe detection |
|  |  |  | laboratory | UMLS:LNC:94565-9 | SARS-CoV-2 (COVID-19) RNA [Presence] in Nasopharynx by NAA with non-probe detection |
|  |  |  | laboratory | UMLS:LNC:95608-6 | SARS-CoV-2 (COVID-19) RNA [Presence] in Respiratory specimen by NAA with non-probe detection |
|  |  |  | laboratory | UMLS:LNC:94759-8 | SARS-CoV-2 (COVID-19) RNA [Presence] in Nasopharynx by NAA with probe detection |
|  |  |  | laboratory | UMLS:LNC:94845-5 | SARS-CoV-2 (COVID-19) RNA [Presence] in Saliva (oral fluid) by NAA with probe detection |
|  |  |  | laboratory | UMLS:LNC:96119-3 | SARS-CoV-2 (COVID-19) Ag [Presence] in Upper respiratory specimen by Immunoassay |
|  |  |  | laboratory | UMLS:LNC:94558-4 | SARS-CoV-2 (COVID-19) Ag [Presence] in Respiratory specimen by Rapid immunoassay |
|  | date constraint | | The terms in this group occurred at any time | | |
|  | event relationship | | Any instance of died occurred within 6 months on or after the first instance of Covid-19 | | |
|  | **Group 4B died** | | | | |
|  | cannot have |  | demographics | Deceased | Deceased |
| Group 5 | | | | | |
|  | **Group 5A Covid-19** | | | | |
|  | must have | any of | diagnosis | UMLS:ICD10CM:U07.1 | COVID-19 |
|  |  |  | diagnosis | UMLS:ICD10CM:J12.81 | Pneumonia due to SARS-associated coronavirus |
|  |  |  | diagnosis | UMLS:ICD10CM:J12.82 | Pneumonia due to coronavirus disease 2019 |
|  |  |  | laboratory | TNX:9088 | SARS coronavirus 2 and related RNA [Presence] |
|  |  |  | laboratory | UMLS:LNC:94309-2 | SARS-CoV-2 (COVID-19) RNA [Presence] in Specimen by NAA with probe detection |
|  |  |  | laboratory | UMLS:LNC:94500-6 | SARS-CoV-2 (COVID-19) RNA [Presence] in Respiratory specimen by NAA with probe detection |
|  |  |  | laboratory | UMLS:LNC:94502-2 | SARS-related coronavirus RNA [Presence] in Respiratory specimen by NAA with probe detection |
|  |  |  | laboratory | UMLS:LNC:95406-5 | SARS-CoV-2 (COVID-19) RNA [Presence] in Nose by NAA with probe detection |
|  |  |  | laboratory | UMLS:LNC:94565-9 | SARS-CoV-2 (COVID-19) RNA [Presence] in Nasopharynx by NAA with non-probe detection |
|  |  |  | laboratory | UMLS:LNC:95608-6 | SARS-CoV-2 (COVID-19) RNA [Presence] in Respiratory specimen by NAA with non-probe detection |
|  |  |  | laboratory | UMLS:LNC:94759-8 | SARS-CoV-2 (COVID-19) RNA [Presence] in Nasopharynx by NAA with probe detection |
|  |  |  | laboratory | UMLS:LNC:94845-5 | SARS-CoV-2 (COVID-19) RNA [Presence] in Saliva (oral fluid) by NAA with probe detection |
|  |  |  | laboratory | UMLS:LNC:96119-3 | SARS-CoV-2 (COVID-19) Ag [Presence] in Upper respiratory specimen by Immunoassay |
|  |  |  | laboratory | UMLS:LNC:94558-4 | SARS-CoV-2 (COVID-19) Ag [Presence] in Respiratory specimen by Rapid immunoassay |
|  | date constraint | | The terms in this group occurred at any time | | |
|  | event relationship | | Any instance of long covid occurred within 1 year and 14 days before the first instance of Covid-19 | | |
|  | **Group 5B long covid** | | | | |
|  | cannot have |  | diagnosis | UMLS:ICD10CM:R07 | Pain in throat and chest |
|  |  | or | diagnosis | UMLS:ICD10CM:R06 | Abnormalities of breathing |
|  |  | or | diagnosis | UMLS:ICD10CM:R10 | Abdominal and pelvic pain |
|  |  | or | diagnosis | UMLS:ICD10CM:R19.4 | Change in bowel habit |
|  |  | or | diagnosis | UMLS:ICD10CM:R19.7 | Diarrhea, unspecified |
|  |  | or | diagnosis | UMLS:ICD10CM:G93.3 | Postviral and related fatigue syndromes |
|  |  | or | diagnosis | UMLS:ICD10CM:R53 | Malaise and fatigue |
|  |  | or | diagnosis | UMLS:ICD10CM:F30-F39 | Mood [affective] disorders |
|  |  | or | diagnosis | UMLS:ICD10CM:F40-F48 | Anxiety, dissociative, stress-related, somatoform and other nonpsychotic mental disorders |
|  |  | or | diagnosis | UMLS:ICD10CM:R51 | Headache |
|  |  | or | diagnosis | UMLS:ICD10CM:G43 | Migraine |
|  |  | or | diagnosis | UMLS:ICD10CM:G44 | Other headache syndromes |
|  |  | or | diagnosis | UMLS:ICD10CM:R40 | Somnolence, stupor and coma |
|  |  | or | diagnosis | UMLS:ICD10CM:R41 | Other symptoms and signs involving cognitive functions and awareness |
|  |  | or | diagnosis | UMLS:ICD10CM:R48 | Dyslexia and other symbolic dysfunctions, not elsewhere classified |
|  |  | or | diagnosis | UMLS:ICD10CM:G93.40 | Encephalopathy, unspecified |
|  |  | or | diagnosis | UMLS:ICD10CM:G31.84 | Mild cognitive impairment of uncertain or unknown etiology |
|  |  | or | diagnosis | UMLS:ICD10CM:G30 | Alzheimer's disease |
|  |  | or | diagnosis | UMLS:ICD10CM:G31.0 | Frontotemporal dementia |
|  |  | or | diagnosis | UMLS:ICD10CM:G31.83 | Neurocognitive disorder with Lewy bodies |
|  |  | or | diagnosis | UMLS:ICD10CM:F01 | Vascular dementia |
|  |  | or | diagnosis | UMLS:ICD10CM:F02 | Dementia in other diseases classified elsewhere |
|  |  | or | diagnosis | UMLS:ICD10CM:F03 | Unspecified dementia |
|  |  | or | diagnosis | UMLS:ICD10CM:F05 | Delirium due to known physiological condition |
|  |  | or | diagnosis | UMLS:ICD10CM:F06.8 | Other specified mental disorders due to known physiological condition |
|  |  | or | diagnosis | UMLS:ICD10CM:M79.1 | Myalgia |
|  |  | or | diagnosis | UMLS:ICD10CM:M60 | Myositis |
|  |  | or | diagnosis | UMLS:ICD10CM:R43.8 | Other disturbances of smell and taste |
|  |  | or | diagnosis | UMLS:ICD10CM:G47.0 | Insomnia |
|  |  | or | diagnosis | UMLS:ICD10CM:G47.9 | Sleep disorder, unspecified |
|  |  | or | diagnosis | UMLS:ICD10CM:R05 | Cough |
|  |  | or | diagnosis | UMLS:ICD10CM:R00.2 | Palpitations |
|  |  | or | diagnosis | UMLS:ICD10CM:U09.9 | Post COVID-19 condition, unspecified |

## Analysis Setup

This section contains the Index Event and Time Window definitions and a list of selected outcomes and the analyses.

### Index Event & Time Window Definitions

The index event defines the point in time when each patient in the cohort enters the analysis. To define an index event for the cohort, one or more criteria for the cohort must be selected. The index date for each patient within a cohort is the day on which the patient first met the selected criteria for the cohort (listed in the table below).

As the index event defines the earliest time point after which outcomes are analyzed, the time window defines the duration during which outcomes are analyzed. The time window can start on the same day as the index event or at any specified time interval after the index event. The time window can end any time after the start date. Outcomes are defined as diagnoses, medications, procedures, or laboratory values that happened in the time window starting after the first occurrence of the index event.

### Time Window Used in this Analysis

This analysis included outcomes that occurred in the time window that started 90 days after the first occurrence of the index event and ended 180 days after the first occurrence of the index event

The index event only includes events that occurred up to 20 years ago. Patients whose index event occurred 20 years or more ago are excluded. In this analysis, 0 patients in Cohort 1 and 0 patients in Cohort 2 were excluded because they met the index event more than 20 years ago.

### **Table S3:** Index Events Used in this Analysis

Index events for the Compare Outcomes analysis were derived from the cohort definitions. Index events were defined separately for each cohort and were based on the criteria used in the original cohort definition.

The index event for Cohort was defined as the following:

|  | | | | | |
| --- | --- | --- | --- | --- | --- |
|  | **Group Covid-19** | | | | |
|  | must have | any of | diagnosis | UMLS:ICD10CM:U07.1 | COVID-19 |
|  |  |  | diagnosis | UMLS:ICD10CM:J12.81 | Pneumonia due to SARS-associated coronavirus |
|  |  |  | diagnosis | UMLS:ICD10CM:J12.82 | Pneumonia due to coronavirus disease 2019 |
|  |  |  | laboratory | TNX:9088 | SARS coronavirus 2 and related RNA [Presence] |
|  |  |  | laboratory | UMLS:LNC:94309-2 | SARS-CoV-2 (COVID-19) RNA [Presence] in Specimen by NAA with probe detection |
|  |  |  | laboratory | UMLS:LNC:94500-6 | SARS-CoV-2 (COVID-19) RNA [Presence] in Respiratory specimen by NAA with probe detection |
|  |  |  | laboratory | UMLS:LNC:94502-2 | SARS-related coronavirus RNA [Presence] in Respiratory specimen by NAA with probe detection |
|  |  |  | laboratory | UMLS:LNC:95406-5 | SARS-CoV-2 (COVID-19) RNA [Presence] in Nose by NAA with probe detection |
|  |  |  | laboratory | UMLS:LNC:94565-9 | SARS-CoV-2 (COVID-19) RNA [Presence] in Nasopharynx by NAA with non-probe detection |
|  |  |  | laboratory | UMLS:LNC:95608-6 | SARS-CoV-2 (COVID-19) RNA [Presence] in Respiratory specimen by NAA with non-probe detection |
|  |  |  | laboratory | UMLS:LNC:94759-8 | SARS-CoV-2 (COVID-19) RNA [Presence] in Nasopharynx by NAA with probe detection |
|  |  |  | laboratory | UMLS:LNC:94845-5 | SARS-CoV-2 (COVID-19) RNA [Presence] in Saliva (oral fluid) by NAA with probe detection |
|  |  |  | laboratory | UMLS:LNC:96119-3 | SARS-CoV-2 (COVID-19) Ag [Presence] in Upper respiratory specimen by Immunoassay |
|  |  |  | laboratory | UMLS:LNC:94558-4 | SARS-CoV-2 (COVID-19) Ag [Presence] in Respiratory specimen by Rapid immunoassay |

### Survival Analysis

The Kaplan-Meier Analysis estimates probability of the outcome at a respective time interval (daily time interval is used in this analysis). In order to account for the patients who exited the cohort during the analysis period, and therefore should not be included in the analysis, censoring is applied. In this analysis, patients are removed from the analysis (censored) after the last fact in their record.

### **Table S4:** Outcome Definitions

Table below outlines the definitions for each outcome and the analysis specifications. For outcome definitions consisting of more than one term, at least one term must match. Please see Appendix C for the text representation of the outcome definitions.

| Any post-covid condition | | | | |
| --- | --- | --- | --- | --- |
|  | **Outcome definition** | | | |
|  | | Diagnosis | UMLS:ICD10CM:R07 | Pain in throat and chest |
|  | | Diagnosis | UMLS:ICD10CM:R06 | Abnormalities of breathing |
|  | | Diagnosis | UMLS:ICD10CM:R10 | Abdominal and pelvic pain |
|  | | Diagnosis | UMLS:ICD10CM:R19.4 | Change in bowel habit |
|  | | Diagnosis | UMLS:ICD10CM:R19.7 | Diarrhea, unspecified |
|  | | Diagnosis | UMLS:ICD10CM:G93.3 | Postviral and related fatigue syndromes |
|  | | Diagnosis | UMLS:ICD10CM:R53 | Malaise and fatigue |
|  | | Diagnosis | UMLS:ICD10CM:F30-F39 | Mood [affective] disorders |
|  | | Diagnosis | UMLS:ICD10CM:F40-F48 | Anxiety, dissociative, stress-related, somatoform and other nonpsychotic mental disorders |
|  | | Diagnosis | UMLS:ICD10CM:R51 | Headache |
|  | | Diagnosis | UMLS:ICD10CM:G43 | Migraine |
|  | | Diagnosis | UMLS:ICD10CM:G44 | Other headache syndromes |
|  | | Diagnosis | UMLS:ICD10CM:R40 | Somnolence, stupor and coma |
|  | | Diagnosis | UMLS:ICD10CM:R41 | Other symptoms and signs involving cognitive functions and awareness |
|  | | Diagnosis | UMLS:ICD10CM:R48 | Dyslexia and other symbolic dysfunctions, not elsewhere classified |
|  | | Diagnosis | UMLS:ICD10CM:F01 | Vascular dementia |
|  | | Diagnosis | UMLS:ICD10CM:F02 | Dementia in other diseases classified elsewhere |
|  | | Diagnosis | UMLS:ICD10CM:F03 | Unspecified dementia |
|  | | Diagnosis | UMLS:ICD10CM:G31.83 | Neurocognitive disorder with Lewy bodies |
|  | | Diagnosis | UMLS:ICD10CM:G31.84 | Mild cognitive impairment of uncertain or unknown etiology |
|  | | Diagnosis | UMLS:ICD10CM:G93.40 | Encephalopathy, unspecified |
|  | | Diagnosis | UMLS:ICD10CM:G30 | Alzheimer's disease |
|  | | Diagnosis | UMLS:ICD10CM:G31.0 | Frontotemporal dementia |
|  | | Diagnosis | UMLS:ICD10CM:F05 | Delirium due to known physiological condition |
|  | | Diagnosis | UMLS:ICD10CM:F06.8 | Other specified mental disorders due to known physiological condition |
|  | | Diagnosis | UMLS:ICD10CM:M79.1 | Myalgia |
|  | | Diagnosis | UMLS:ICD10CM:M60 | Myositis |
|  | | Diagnosis | UMLS:ICD10CM:R43.8 | Other disturbances of smell and taste |
|  | | Diagnosis | UMLS:ICD10CM:G47.0 | Insomnia |
|  | | Diagnosis | UMLS:ICD10CM:G47.9 | Sleep disorder, unspecified |
|  | | Diagnosis | UMLS:ICD10CM:R05 | Cough |
|  | | Diagnosis | UMLS:ICD10CM:R00.2 | Palpitations |
|  | | Diagnosis | UMLS:ICD10CM:U09.9 | Post COVID-19 condition, unspecified |
|  | **Settings for the performed analyses** | | | |
|  | | Kaplan - Meier survival analysis | | including patients with outcome prior to the time window |
| 1) Chest/Throat pain: | | | | |
|  | **Outcome definition** | | | |
|  | | Diagnosis | UMLS:ICD10CM:R07 | Pain in throat and chest |
|  | **Settings for the performed analyses** | | | |
|  | | Kaplan - Meier survival analysis | | including patients with outcome prior to the time window |
| Abnormal breathing | | | | |
|  | **Outcome definition** | | | |
|  | | Diagnosis | UMLS:ICD10CM:R06 | Abnormalities of breathing |
|  | **Settings for the performed analyses** | | | |
|  | | Kaplan - Meier survival analysis | | including patients with outcome prior to the time window |
| Abdominal symptoms | | | | |
|  | **Outcome definition** | | | |
|  | | Diagnosis | UMLS:ICD10CM:R10 | Abdominal and pelvic pain |
|  | | Diagnosis | UMLS:ICD10CM:R19.4 | Change in bowel habit |
|  | | Diagnosis | UMLS:ICD10CM:R19.7 | Diarrhea, unspecified |
|  | **Settings for the performed analyses** | | | |
|  | | Kaplan - Meier survival analysis | | including patients with outcome prior to the time window |
| Fatigue: | | | | |
|  | **Outcome definition** | | | |
|  | | Diagnosis | UMLS:ICD10CM:G93.3 | Postviral and related fatigue syndromes |
|  | | Diagnosis | UMLS:ICD10CM:R53 | Malaise and fatigue |
|  | **Settings for the performed analyses** | | | |
|  | | Kaplan - Meier survival analysis | | including patients with outcome prior to the time window |
| Anxiety/Depression | | | | |
|  | **Outcome definition** | | | |
|  | | Diagnosis | UMLS:ICD10CM:F30-F39 | Mood [affective] disorders |
|  | | Diagnosis | UMLS:ICD10CM:F40-F48 | Anxiety, dissociative, stress-related, somatoform and other nonpsychotic mental disorders |
|  | **Settings for the performed analyses** | | | |
|  | | Kaplan - Meier survival analysis | | including patients with outcome prior to the time window |
| headache | | | | |
|  | **Outcome definition** | | | |
|  | | Diagnosis | UMLS:ICD10CM:R51 | Headache |
|  | | Diagnosis | UMLS:ICD10CM:G43 | Migraine |
|  | | Diagnosis | UMLS:ICD10CM:G44 | Other headache syndromes |
|  | **Settings for the performed analyses** | | | |
|  | | Kaplan - Meier survival analysis | | including patients with outcome prior to the time window |
| Cognitive symptoms | | | | |
|  | **Outcome definition** | | | |
|  | | Diagnosis | UMLS:ICD10CM:R40 | Somnolence, stupor and coma |
|  | | Diagnosis | UMLS:ICD10CM:R41 | Other symptoms and signs involving cognitive functions and awareness |
|  | | Diagnosis | UMLS:ICD10CM:R48 | Dyslexia and other symbolic dysfunctions, not elsewhere classified |
|  | | Diagnosis | UMLS:ICD10CM:G93.40 | Encephalopathy, unspecified |
|  | | Diagnosis | UMLS:ICD10CM:G31.84 | Mild cognitive impairment of uncertain or unknown etiology |
|  | | Diagnosis | UMLS:ICD10CM:G30 | Alzheimer's disease |
|  | | Diagnosis | UMLS:ICD10CM:G31.0 | Frontotemporal dementia |
|  | | Diagnosis | UMLS:ICD10CM:G31.83 | Neurocognitive disorder with Lewy bodies |
|  | | Diagnosis | UMLS:ICD10CM:F01 | Vascular dementia |
|  | | Diagnosis | UMLS:ICD10CM:F02 | Dementia in other diseases classified elsewhere |
|  | | Diagnosis | UMLS:ICD10CM:F03 | Unspecified dementia |
|  | | Diagnosis | UMLS:ICD10CM:F05 | Delirium due to known physiological condition |
|  | | Diagnosis | UMLS:ICD10CM:F06.8 | Other specified mental disorders due to known physiological condition |
|  | **Settings for the performed analyses** | | | |
|  | | Kaplan - Meier survival analysis | | including patients with outcome prior to the time window |
| 9) Myalgia | | | | |
|  | **Outcome definition** | | | |
|  | | Diagnosis | UMLS:ICD10CM:M79.1 | Myalgia |
|  | | Diagnosis | UMLS:ICD10CM:M60 | Myositis |
|  | **Settings for the performed analyses** | | | |
|  | | Kaplan - Meier survival analysis | | including patients with outcome prior to the time window |
| Loss of taste/smell: | | | | |
|  | **Outcome definition** | | | |
|  | | Diagnosis | UMLS:ICD10CM:R43.8 | Other disturbances of smell and taste |
|  | **Settings for the performed analyses** | | | |
|  | | Kaplan - Meier survival analysis | | including patients with outcome prior to the time window |
| Sleep disturbance: | | | | |
|  | **Outcome definition** | | | |
|  | | Diagnosis | UMLS:ICD10CM:G47.0 | Insomnia |
|  | | Diagnosis | UMLS:ICD10CM:G47.9 | Sleep disorder, unspecified |
|  | **Settings for the performed analyses** | | | |
|  | | Kaplan - Meier survival analysis | | including patients with outcome prior to the time window |
| Cough: | | | | |
|  | **Outcome definition** | | | |
|  | | Diagnosis | UMLS:ICD10CM:R05 | Cough |
|  | **Settings for the performed analyses** | | | |
|  | | Kaplan - Meier survival analysis | | including patients with outcome prior to the time window |
| Palpitation | | | | |
|  | **Outcome definition** | | | |
|  | | Diagnosis | UMLS:ICD10CM:R00.2 | Palpitations |
|  | **Settings for the performed analyses** | | | |
|  | | Kaplan - Meier survival analysis | | including patients with outcome prior to the time window |
| 15) U09.9 Post COVID-19 condition | | | | |
|  | **Outcome definition** | | | |
|  | | Diagnosis | UMLS:ICD10CM:U09.9 | Post COVID-19 condition, unspecified |
|  | **Settings for the performed analyses** | | | |
|  | | Kaplan - Meier survival analysis | | including patients with outcome prior to the time window |

Figure S1. Forest plots of primary outcome and its components stratified by sex.


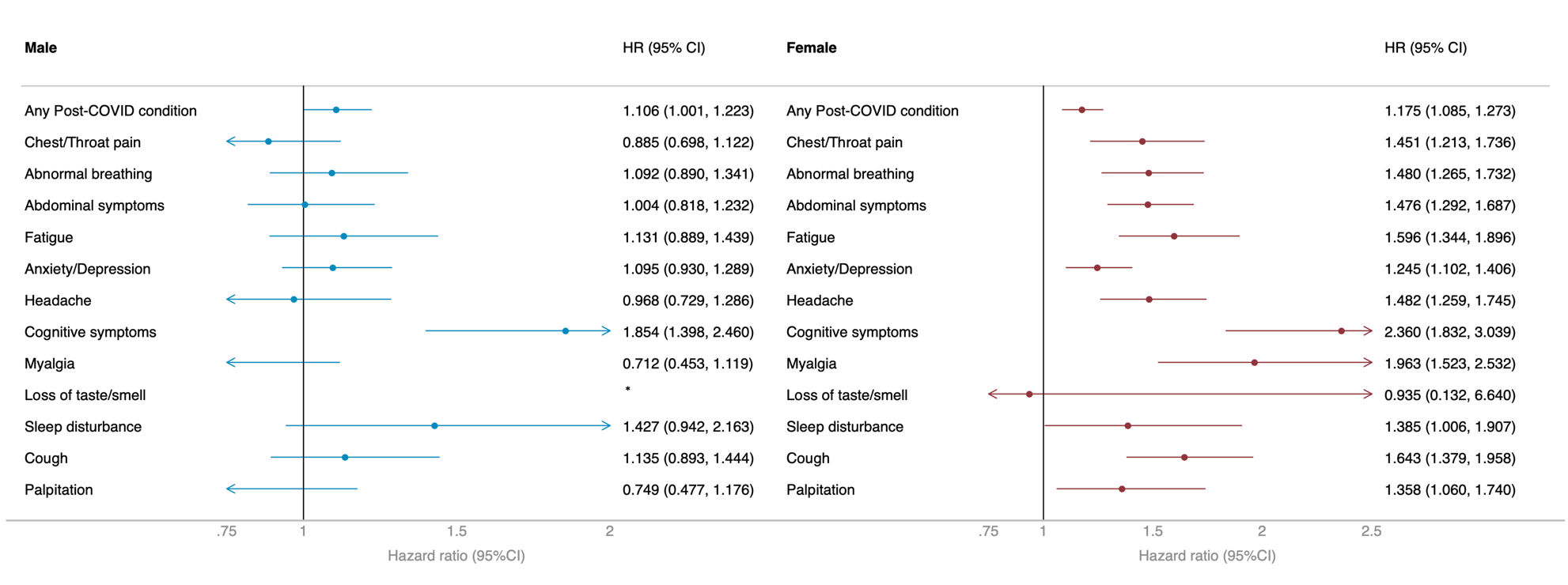


Figure S2. Forest plots of primary outcome and its components stratified by age.


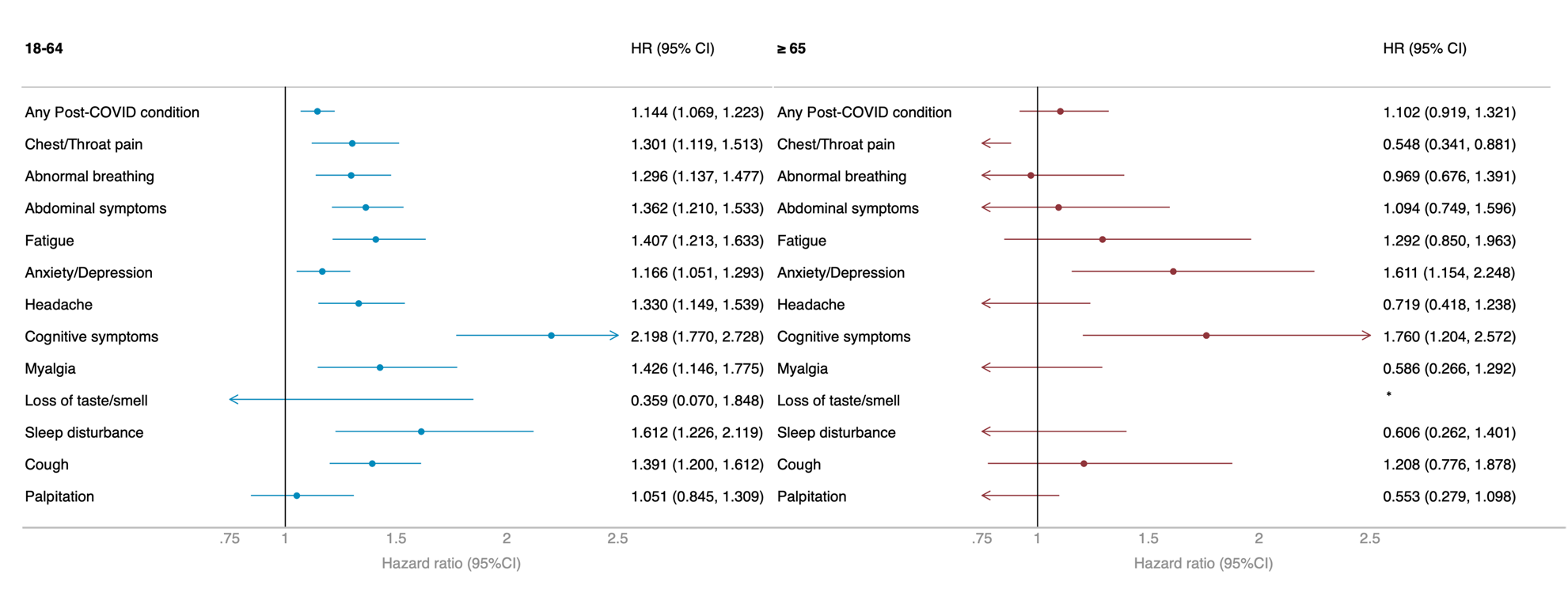


Figure S3. Forest plots of primary outcome and its components stratified by vaccine status.


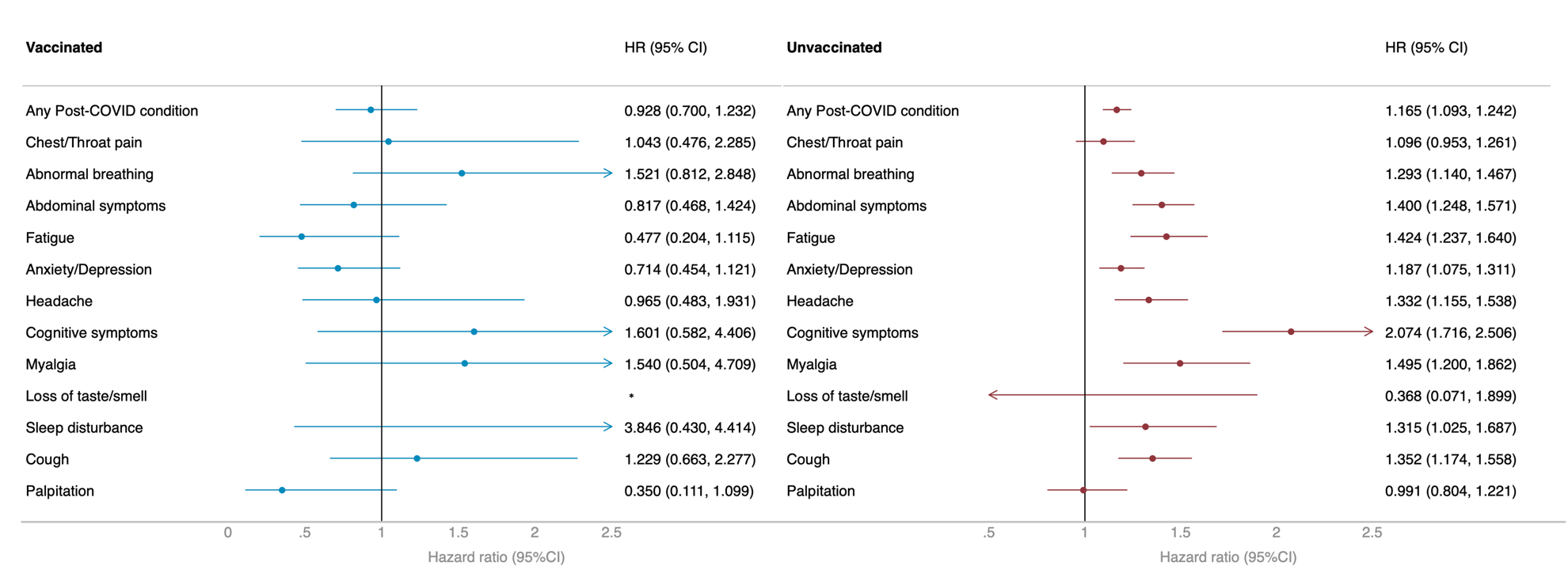


Figure S4. Forest plots of primary outcome and its components stratified by race


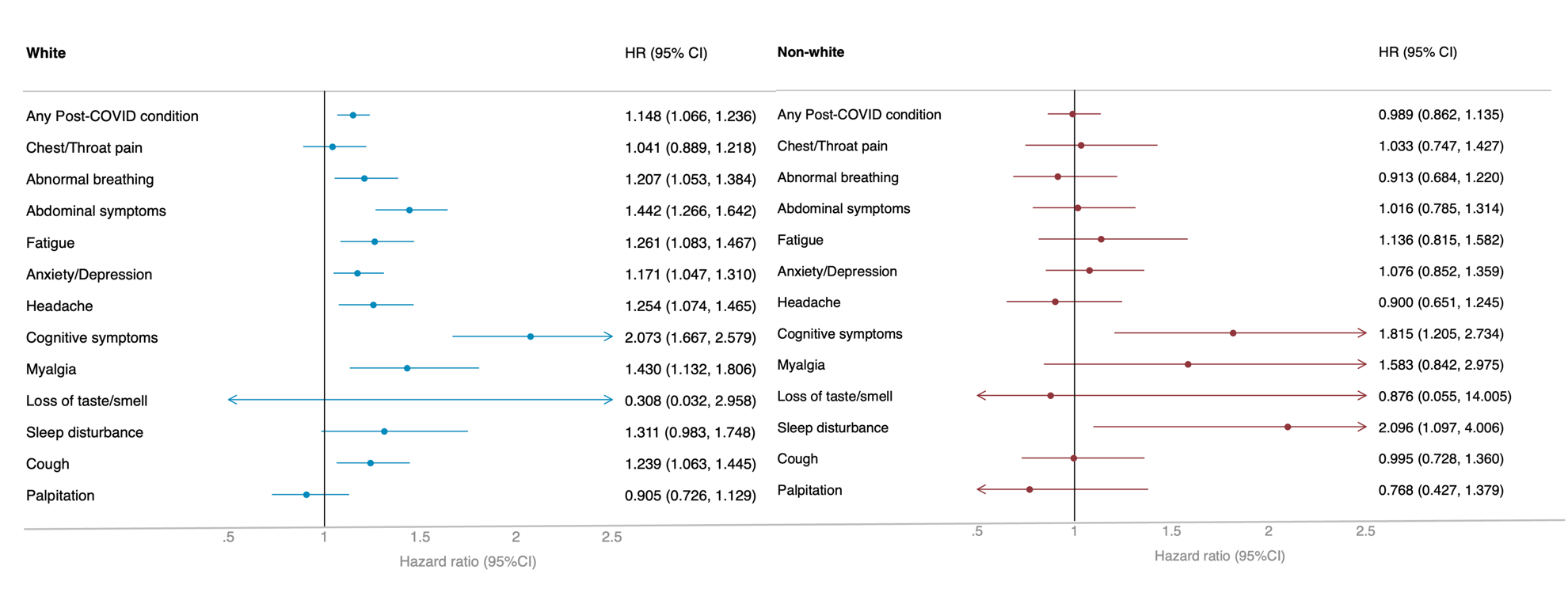

Supplement: Supplementary file 1 — Additional file 1: Table S1. Query Criteria for Cohort (query name: IDD). Table S2. Query Criteria for Cohort (query name: W/O IDD). Table S3. Index Events Used in this Analysis. Table S4. Outcome Definitions. Fig. S1. Forest plots of primary outcome and its components stratified by sex. Fig. S2. Forest plots of primary outcome and its components stratified by age. Fig. S3. Forest plots of primary outcome and its components stratified by vaccine status. Fig. S4. Forest plots of primary outcome and its components stratified by race. [file 12916_2023_3216_MOESM1_ESM.docx]
